# Impact of individual extracellular proteases on *Staphylococcus aureus* biofilm formation in diverse clinical isolates and their isogenic *sarA* mutants

**DOI:** 10.1002/mbo3.214

**Published:** 2014-09-25

**Authors:** Allister J Loughran, Danielle N Atwood, Allison C Anthony, Nada S Harik, Horace J Spencer, Karen E Beenken, Mark S Smeltzer

**Affiliations:** 1 Department of Microbiology and Immunology, University of Arkansas for Medical SciencesLittle Rock, Arkansas; 2 Department of Pediatrics, Arkansas Children's HospitalLittle Rock, Arkansas; 3 Department of Biostatistics, University of Arkansas for Medical SciencesLittle Rock, Arkansas; 4 Department of Orthopaedic Surgery, University of Arkansas for Medical SciencesLittle Rock, Arkansas; 5 Department of Pathology, University of Arkansas for Medical SciencesLittle Rock, Arkansas

**Keywords:** Biofilm, proteases, *Staphylococcus aureus*, *sarA*

## Abstract

We demonstrate that the purified *Staphylococcus aureus* extracellular proteases aureolysin, ScpA, SspA, and SspB limit biofilm formation, with aureolysin having the greatest impact. Using protease-deficient derivatives of LAC, we confirmed that this is due to the individual proteases themselves. Purified aureolysin, and to a lesser extent ScpA and SspB, also promoted dispersal of an established biofilm. Mutation of the genes encoding these proteases also only partially restored biofilm formation in an FPR3757 *sarA* mutant and had little impact on restoring virulence in a murine bacteremia model. In contrast, eliminating the production of all of these proteases fully restored both biofilm formation and virulence in a *sarA* mutant generated in the closely related USA300 strain LAC. These results confirm an important role for multiple extracellular proteases in *S. aureus* pathogenesis and the importance of *sarA* in repressing their production. Moreover, purified aureolysin limited biofilm formation in 14 of 15 methicillin-resistant isolates and 11 of 15 methicillin-susceptible isolates, while dispersin B had little impact in UAMS-1, LAC, or 29 of 30 contemporary isolates of *S. aureus*. This suggests that the role of *sarA* and its impact on protease production is important in diverse strains of *S. aureus* irrespective of their methicillin resistance status.

## Introduction

Mutation of the staphylococcal accessory regulator (*sarA*) attenuates the virulence of *Staphylococcus aureus* in murine models of both bacteremia and catheter-associated biofilm formation (Blevins et al. 2003; Weiss et al. 2009a, 2009b; Zielinska et al. 2012). Mutation of *sarA* also results in the increased production of all 10 recognized *S. aureus* extracellular proteases (aureolysin, the serine proteases SspA and SplA-F, and the cysteine proteases ScpA and SspB), and in the USA300 strain LAC this has been correlated with the reduced capacity of a *sarA* mutant to cause disease in both of these models (Zielinska et al. 2012). Mutation of *saeRS* in LAC was also shown to result in an overall increase in proteolytic activity (Mrak et al. 2012), and a recent paper confirmed that this includes increased production of aureolysin, SspA, and SspB but not the *spl*-encoded proteases (Cassat et al. 2013). The LAC *saeRS* mutant also exhibited reduced virulence in a murine osteomyelitis model, and mutation of the gene encoding aureolysin (*aur*) reversed this effect (Cassat et al. 2013).

*Staphylococcus aureus* proteases are known to degrade components of host defense systems including complement (Jusko et al. 2014). However, it seems unlikely that this plays a primary role in explaining the in vivo phenotypes discussed above because, if this were the case, the increased production of proteases in *sarA* and *saeRS* mutants would be expected to limit host defenses and thereby increase, rather than decrease, the capacity to cause infection. Thus, the more likely explanation is that the increased production of extracellular proteases in these mutants limits the accumulation of *S. aureus* virulence factors. Under in vitro conditions, we confirmed that the increased production of extracellular proteases in *sarA* mutants can be correlated with reduced accumulation of over 250 *S. aureus* proteins including the fibronectin-binding protein FnbA, protein A (Spa), alpha toxin, and phenol-soluble modulins (PSMs) (Zielinska et al. 2011, 2012; Mrak et al. 2012). All of which have been implicated in various aspects of *S. aureus* pathogenesis including biofilm formation (Caiazza and O'Toole 2003; Merino et al. 2009; Houston et al. 2011; Schwartz et al. 2012). Similarly, Cassat et al. (2013) found that, of 269 proteins that decrease in abundance in an *saeRS* mutant, the abundance of 225 (83%) is at least partially restored by concomitant mutation of the gene encoding *aur*, with 190 of these returning to levels that meet or exceed those observed in the parent strain.

Our own studies have demonstrated that FnbA accumulation can be restored in *sarA* mutants by mutation of the *sspABC* operon, while accumulation of Spa, alpha toxin, and PSMs can be restored by mutation of the gene encoding *aur* (Zielinska et al. 2011; Mrak et al. 2012). Restoration of PSM accumulation in a *saeRS/aur* mutant was also confirmed by Cassat et al. (2013). Aureolysin is required for full activation of SspA, which is in turn required for full activation of SspB (Shaw et al. 2004), leaving open the possibility that the impact of aureolysin on accumulation of these virulence factors is indirect. However, mutation of the *sspABC* operon in *sarA* mutants had little impact on the accumulation of Spa, alpha toxin, or PSMs, suggesting that aureolysin itself is responsible for these effects (Zielinska et al. 2011; Mrak et al. 2012).

A recent report also demonstrated that eliminating production of the cysteine proteases ScpA and SspB, both of which are produced in increased amounts in *sarA* and *saeRS* mutants (Zielinska et al. 2012; Cassat et al. 2013), restored biofilm formation in a LAC *sigB* mutant and, in the case of ScpA, promote dispersal of biofilms formed by LAC itself (Mootz et al.*,* 2013). The biofilm assays used in this report included a plasma-coated substrate, and it was suggested that these effects were likely due to degradation of both *S. aureus* adhesins and host protein targets of these adhesins. In an independent report, mutation of *sigB* was also correlated with increased expression of the accessory gene regulator (*agr*) and a decreased capacity to form a biofilm; in this case, restoration of biofilm formation required concomitant mutation of the genes encoding both aureolysin and the *spl*-encoded proteases (Lauderdale et al. 2009).

The six *spl*-encoded proteases are the only ones that are not highly conserved among diverse clinical isolates. One study found that 31% of strains encoded the entire *spl* operon, with 16% lacking the entire operon (Zdzalik et al. 2012). The prevalence of the six genes in the remaining 53% of the strains examined ranged from 54% (*splD*) to 92% (*splC*). At the same time, antibody titers to all *spl*-encoded proteases were shown to be higher in infected than uninfected patients. In infected patients antibody, titers to the *spl*-encoded proteases were higher than those to all other extracellular proteases (Zdzalik et al. 2012). These results demonstrate that, when present, the *spl*-encoded proteases are produced in vivo. Therefore, all 10 recognized extracellular proteases have been implicated as limiting factors in *S. aureus* biofilm formation under in vitro if not in vivo conditions.

When taken together, these results confirm an important role for extracellular proteases in multiple forms of *S. aureus* infection including the transition to a biofilm mode of growth. This implies that extracellular proteases could potentially be exploited to therapeutic advantage, particularly in the context of biofilm-associated infections. However, many of the studies done to date have focused on the methicillin-resistant USA300 strain LAC, which is potentially important in that it has been suggested that surface proteins, most notably FnbA and FnbB, play a more important role in methicillin-resistant *S. aureus* strains (MRSA) than in methicillin-susceptible strains (MSSA), with the latter relying more heavily on the polysaccharide intercellular adhesin (PIA) (O'Neill et al. 2008; Houston et al. 2011; Pozzi et al. 2012). Indeed, in the laboratory strain 8325-4, production of the *mecA*-encoded penicillin-binding protein PBP2a was found to repress expression of the *ica* operon and thereby limit the production of PIA, thus suggesting a direct cause-and-effect relationship between methicillin resistance status and the mechanism of biofilm formation (Pozzi et al. 2012). This suggests that therapeutic strategies focusing on protease production, or proteases themselves (Vandecandelaere et al. 2014), would have limited utility in the treatment of biofilm-associated infections caused by MSSA strains. It should be noted, in this respect, that while infections caused by USA300 strains like LAC are often highly invasive (Wang et al.*,* 2007), this does not preclude the need to address infections caused by MSSA strains, particularly in the specific context of chronic biofilm-associated infections.

Our studies demonstrate that mutation of *sarA* limits biofilm formation in diverse clonal lineages irrespective of methicillin resistance status and that this is due to the increased production of extracellular proteases (Tsang et al. 2008; Weiss et al. 2009a, 2009b; Beenken et al. 2012; Mrak et al. 2012). However, these studies have been limited to a relatively small number of strains, most notably the MSSA strains UAMS-1 and the USA300, MRSA strains LAC and FPR3757. Additionally, our most critical in vivo experiments focusing on extracellular proteases have been limited to derivatives of LAC unable to produce any of the 10 extracellular proteases (Zielinska et al. 2012; Beenken et al. 2014). Thus, the relative contribution of individual proteases, particularly under in vivo conditions, remains unclear. The primary goal of the experiments we report was to address this issue under both in vitro and in vivo conditions and, secondarily, to more globally examine the extent to which the impact of proteases on biofilm formation differs as a function of methicillin resistance status.

## Experimental Procedures

### Bacterial strains and growth conditions

The *S. aureus* strains utilized in this study included a plasmid cured, erythromycin-sensitive derivative of the MRSA USA300 strains LAC (Wörmann et al. 2011), the closely related USA300 strain FPR3757 (Diep et al. 2006), the USA200 MSSA osteomyelitis isolate UAMS-1 (Cassat et al. 2006), and derivatives of each of these parent strains carrying mutations in *sarA* and/or the genes/operons encoding extracellular proteases (Table 1). Mutants were generated by Φ11-mediated transduction from mutants already on hand or obtained from the Nebraska Transposon Mutant Library maintained at the Network on Antimicrobial Resistance in *S. aureus* (NARSA, http://www.narsa.net). The latter were produced in the JE2 derivative of LAC and were first transduced into our version of LAC (Zielinska et al. 2012) prior to analysis. Because all of the mutants in this library are defined by resistance to erythromycin, in those cases in which it was necessary to mutate more than the protease gene/operon, Φ11-mediated transduction was first used to exchange one or more of the erythromycin resistance cassettes with an alternative antibiotic resistance cassette (Bose et al. 2013). We also employed 30 contemporary clinical isolates obtained from and Arkansas Children's Hospital database, collected over the last 15 years, from nonrelated patients, all of which were shown to be distinct by comparison to each other based on 16 genotypic markers or phenotypic characteristics (data not shown). The *Staphylococcus epidermidis* strain RP62A was included as a control in some experiments because it is known to produce a PIA-dependent biofilm (Gill et al. 2005). Strains were maintained at −80°C in tryptic soy broth (TSB) containing 25% (v/v) glycerol. For analysis, strains were cultured from cold storage by plating on tryptic soy agar (TSA) with the appropriate antibiotic selection. Antibiotics were used at the following concentrations: spectinomycin (Spec; 1000 *μ*g mL^−1^), erythromycin (Erm; 10 *μ*g mL^−1^), kanamycin (Kan; 50 *μ*g mL^−1^), and neomycin (Neo; 50 *μ*g mL^−1^).

**Table 1:** Bacterial strains used in this study.

Strain	Description	Reference
UAMS-1	MSSA, osteomyelitis isolate	Smeltzer et al. (1997)
UAMS-929	UAMS-1, *sarA*::Kan/Neo	Blevins et al. (2002)
UAMS-1794	Erm sensitive FPR3757	Diep et al. (2006)
UAMS-1802	UAMS-1794, *sarA*::Kan/Neo	Zielinska et al. (2011)
UAMS-2279	Erm sensitive LAC	Wörmann et al. (2011)
UAMS-2294	UAMS-2279, *sarA*::Kan/Neo	Zielinska et al. (2012)
UAMS-3001	UAMS-2279, *aur/ssp/scp/spl*::Erm	Wörmann et al. (2011)
UAMS-3002	UAMS-3001, *sarA*::Kan/Neo	Zielinska et al. (2012)
UAMS-4191	UAMS-2279, *aur/ssp/scp*	Beenken et al. (2014)
UAMS-1037	*Staphylococcus epidermidis* RP62A	Gill et al. (2005)
UAMS-4207	UAMS-1802, *aur*::Spec	This study
UAMS-4279	UAMS-1802, *sspA*::Erm	This study
UAMS-4280	UAMS-1802, *sspB*::Erm	This study
UAMS-2206	UAMS-1802, *scp*::Erm	Zielinska et al. (2011)
UAMS-2219	UAMS-1802, *spl*::Erm	Zielinska et al. (2011)
UAMS-2223	UAMS-1802, *sspABC*::Erm	Zielinska et al. (2011)
UAMS-1681	MRSA, leg abscess isolate	ACH 1
UAMS-1592	MRSA, wound isolate	ACH
UAMS-1667	MRSA, blood, femur isolate	ACH
UAMS-1572	MRSA, wound, blood isolate	ACH
UAMS-1692	MRSA, blood, joint isolate	ACH
UAMS-1672	MSSA, blood, knee joint, distal femur isolate	ACH
UAMS-1673	MSSA, blood, urine, retropharyngeal isolate	ACH
UAMS-1688	MSSA, blood, muscle, bone isolate	ACH
UAMS-1743	MSSA, blood, bone isolate	ACH
UAMS-1746	MSSA, blood, abscess isolate	ACH
UAMS-1578	MRSA, blood, joint, bone, wound isolate	ACH
UAMS-1676	MRSA, blood isolate	ACH
UAMS-1683	MRSA, joint, blood, bone isolate	ACH
UAMS-1687	MRSA, blood, joint, bone isolate	ACH
UAMS-1694	MRSA, blood isolate	ACH
UAMS-1579	MSSA, blood isolate	ACH
UAMS-1577	MSSA, blood, joint, wound isolate	ACH
UAMS-1582	MSSA, hip joint, wound isolate	ACH
UAMS-1665	MSSA, ankle, tibia, blood isolate	ACH
UAMS-1670	MSSA, blood, iliacus abscess isolate	ACH
UAMS-1741	MRSA, blood, bone, joint isolate	ACH
UAMS-1745	MRSA, blood isolate	ACH
UAMS-1747	MRSA, blood, pleural fluid, wound isolate	ACH
UAMS-1748	MRSA, blood, joint isolate	ACH
UAMS-1749	MRSA, blood, joint, BAL, wound, bone isolate	ACH
UAMS-1684	MSSA, blood, joint, bone isolate	ACH
UAMS-1690	MSSA, blood, abscess isolate	ACH
UAMS-1691	MSSA, blood, joint, CSF isolate	ACH
UAMS-1695	MSSA, blood, joint isolate	ACH
UAMS-1696	MSSA, joint fluid isolate	ACH

1 Denotes primary clinical isolate obtained from a patient at Arkansas Children's Hospital. Associated description of these strains indicates methicillin-resistance status and site(s) from which the isolate was obtained.

### Extracellular protease activity

Protease activity was assessed with purified proteases (BioCentrum, Krakow, Poland) or standardized samples of stationary phase (16 h) conditioned medium by 10% zymogram (gelatin) gels (Life Technologies, Grand Island, NY) as previously described (Zielinska et al. 2012). Briefly, purified proteases were diluted from 10 *μ*mol/L stock in phosphate buffered saline (1X PBS) to a final concentration of 250 nmol/L, the highest concentration used in any of the experiments contained in this paper. After electrophoresis, gels were incubated at room temperature (RT) for 30 min in renaturing buffer (2.5% Triton X-100) and then overnight at 37°C in developing buffer (0.2 mol/L Tris, 5 mmol/L CaCl_2_, 1 mmol/L Dithiothreitol (DTT)). Gels were then stained with SimplyBlue SafeStrain (Life Technologies) at RT for 2 h before destaining overnight in distilled water.

### Biofilm assay

Biofilm formation was assessed in vitro as previously described (Beenken et al. 2010). Briefly, 96 *μ*L plate wells were first coated with 20% human plasma before growing individual strains for 24 h in TSB supplemented with salt and glucose (biofilm medium, BFM). To assess inhibition of biofilm formation, purified aureolysin, SspA, SspB, or ScpA were added to BFM at the beginning of the experiment. Experiments focusing specifically on aureolysin were assayed using a concentration of 62.5 nmol/L. To assess the impact of Dispersin B (Kane Biotech Inc, Winnipeg, Manitoba, Canada), the enzyme was added to BFM at a concentration of 5 *μ*mol/L (0.2 mg mL^−1^) as previously described (Donelli et al. 2007; Sugimoto et al. 2013). For experiments focusing on biofilm dispersal, biofilms were allowed to form for 24 h before replacing the BFM with BFM containing the appropriate protease. In both cases, the protease concentrations employed ranged from 16 to 250 nmol/L as previously described (Mootz et al. 2013).

### Bacteremia model

Five- to eight-week-old female, outbred NIH-Swiss mice (Harlan, Indianapolis, IN) were infected via tail vein injection with 10^8^ cfu of the indicated strains (Blevins et al. 2003). Experiments were carried out with 10 mice per experimental group. Mice were weighed prior to injection and prior to euthanasia at 6 days post-infection. Immediately after euthanasia, the heart, spleen, and right kidney were harvested for analysis; in those cases in which compassionate euthanasia was required, tissues were harvested immediately after death. For analysis of soft tissue samples, the targeted organ was removed aseptically and homogenized on ice. Dilutions of each homogenate were then plated on TSA with appropriate antibiotic selection for quantitative analysis. The number of cfu per organ was then determined following overnight incubation at 37°C.

### Statistical analysis

Both the in vitro biofilm data and the in vivo bacterial count data were logarithmically transformed prior to analysis and one-way analysis of variance (ANOVA) models were used to analyze the data. For the in vitro biofilm data, traditional *F*-tests were performed to evaluate the overall model while the Tukey–Kramer procedure was used to perform pair-wise testing among mutation types. Because a large number of zero counts were observed in the in vivo bacterial count data, permutation methods were used to determine the significance of the overall ANOVA model as well as for the pair-wise tests, which were performed using *t*-tests. Statistical analyses were performed using R (version 2.7; The Foundation for Statistical Computing http://www.r-project.org/foundation/), SigmaPlot, San Jose, CA, USA and GraphPad Prism 5.0 La Jolla, CA USA. *P*-values ≤0.05 were considered to be statistically significant.

## Results and Discussion

**Figure 1:**
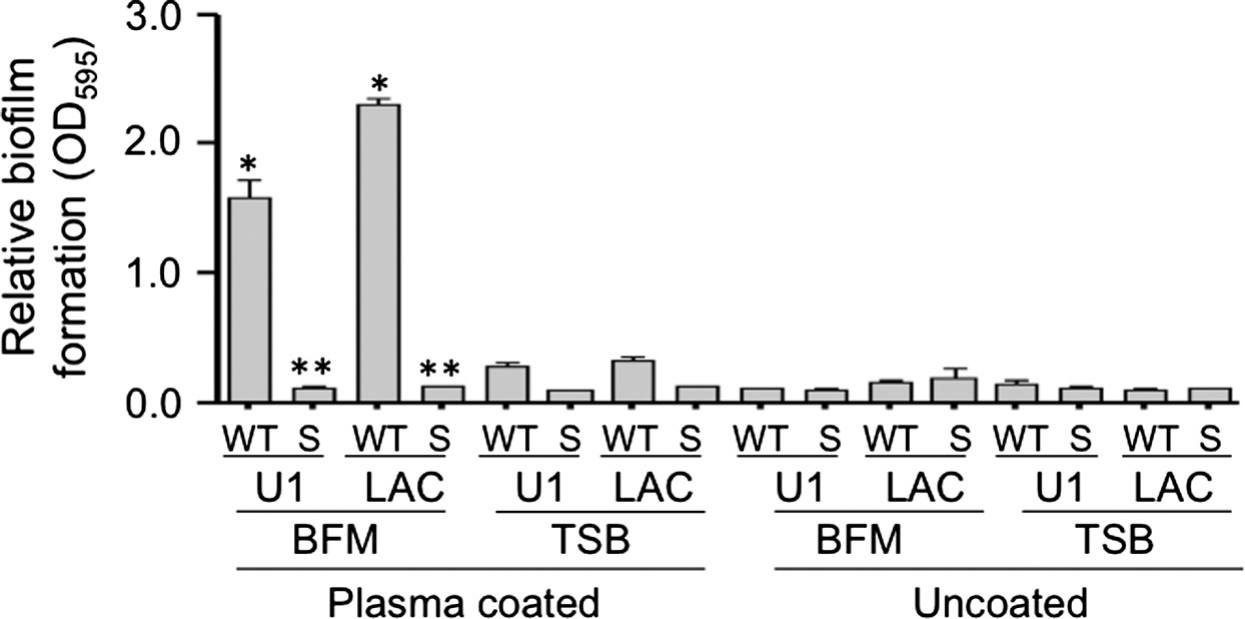
Defining optimal conditions for biofilm formation in vitro. Biofilm formation was assessed in the wild-type (WT) strains UAMS-1 (U1), LAC, and their isogenic *sarA* mutants (S) using tryptic soy broth (TSB) or TSB supplemented with salt and glucose (biofilm media, BFM) with and without first coating the substrate with human plasma. Single asterisk indicates statistical significance associated with growth conditions. Double asterisk indicates statistical significance of the *sarA* mutant by comparison to its isogenic parent strain under these optimized conditions.

**Figure 2:**
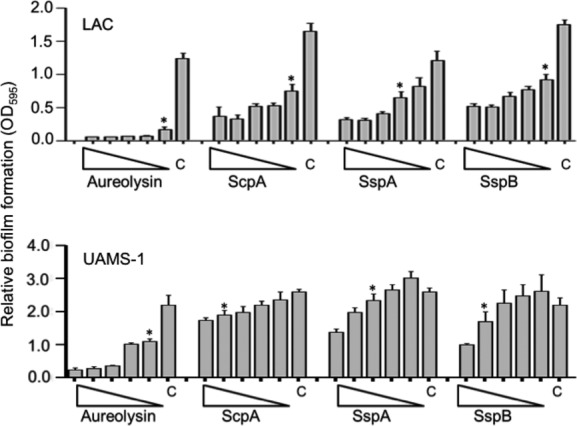
Impact of purified extracellular proteases in LAC (MRSA) and UAMS-1 (MSSA). Purified aureolysin, the serine protease SspA, and the cysteine proteases ScpA or SspB were added individually to BFM prior to initiation of the biofilm assay. The strains used are indicated in each panel. Triangles indicate decreasing concentrations of each protease from 250 to 16 nmol/L, with “C” indicating the control assay without exogenous protease. Asterisks indicate the lowest concentration of each protease at which a statistically significant difference was observed relative to the control.

**Figure 3:**
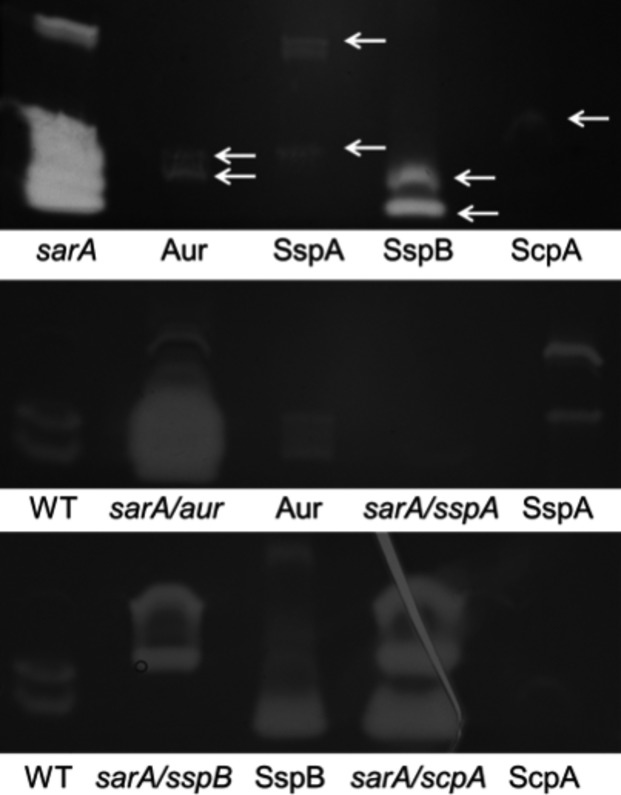
Impact of mutating individual protease genes/operons. Proteolytic activity was assessed in FPR3757 (WT) with and without addition of the indicated protease, its *sarA* mutant, and derivatives of the *sarA* mutant with mutations inactivating the indicated protease genes. Top panel: To ensure the use of physiologically relevant amounts of purified proteases in the context of the amounts produced by the isogenic *sarA* mutant, each purified protease was examined individually at a concentration of 250 nmol/L, which was the highest concentration used in our protease add-back experiments. Bottom panels: The protease phenotype of the WT strain was compared to that of its *sarA* mutant carrying mutations in the indicated protease genes. As discussed in the text, the *sspA* mutation is polar, thus eliminating production of both SspA and SspB. Purified proteases were also included as additional controls.

Given our interest in orthopedic infection and the role of biofilms in these infections (Brady et al. 2008), we initially focused our efforts investigating biofilm formation of the USA200, CC30, MSSA strain UAMS-1, which was isolated from the bone of an osteomyelitis patient during surgical debridement (Smeltzer et al. 1997). Under in vitro conditions, consistent biofilm formation with this strain, as well as almost all others we examined, was dependent on supplementation of the medium with salt and glucose and coating the substrate with human plasma (Beenken et al. 2003). Here, we confirm that this is also true with the USA300, CC8, MRSA strain LAC and that, in both of these strains, biofilm formation under these optimized in vitro conditions is dramatically reduced by mutation of *sarA* (Fig. 1). To the extent that plasma coating had the most dramatic impact on promoting biofilm formation in both of these strains, which were chosen because they are phenotypically and genetically distinct by comparison to each other including their methicillin resistance status (Cassat et al. 2005, 2006), this suggests the existence of a common, protein-dependent mechanism of biofilm formation in diverse contemporary clinical isolates of *S. aureus*. Based on this, we examined the impact of adding purified aureolysin, ScpA, SspA, and SspB (BioCentrum) on preventing biofilm formation. Results confirmed that all four of these proteases limit biofilm formation in both LAC (MRSA) and UAMS-1 (MSSA) (Fig. 2). We also confirmed by zymogram that the amount of each protease required to achieve this effect is lower than the amounts observed in the isogenic *sarA* mutant (Fig. 3), thus suggesting that these experiments are likely to be physiologically relevant at least in the context of defining the biofilm-deficient phenotype of the respective *sarA* mutants.

**Figure 4:**
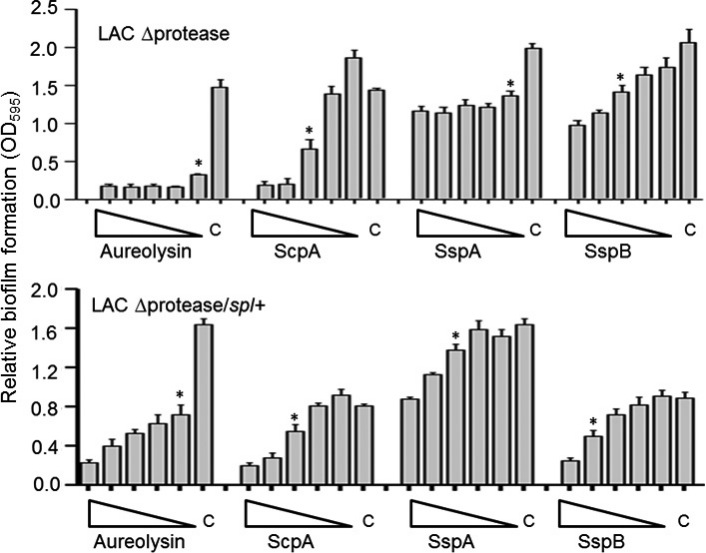
Impact of purified extracellular proteases in LAC protease-deficient derivatives. The indicated proteases were added to the BFM used in biofilm assays using derivatives of LAC unable to produce any extracellular protease (LAC *Δ*protease) or unable to produce any extracellular protease other than those encoded within the *spl* operon (LAC *Δ*protease/*spl*+). Triangles indicate decreasing concentrations from 250 to 16 nmol/L, with “C” indicating the control assay without the addition of any exogenous protease. Asterisks indicate the lowest concentration of each protease at which a statistically significant difference was observed relative to this control.

Although all four of these proteases had an inhibitory effect, the impact of each individual protease was not equivalent, with equivalent molar amounts of aureolysin having the greatest inhibitory effect in both UAMS-1 and LAC (Fig. 2). However, the position of aureolysin at the top of the *S. aureus* protease activation cascade (Shaw et al. 2004; Mootz et al. 2013) leaves open the possibility that aureolysin is not more active but rather that its absence simply decreases the activity of the downstream proteases SspA and/or SspB. To address this, we repeated the experiments using a derivative of LAC unable to produce any of the recognized extracellular proteases. The results confirmed the inhibitory effect of aureolysin, ScpA, SspA, and SspB, but even under these circumstances aureolysin was found to have the greatest relative effect (i.e., the greatest inhibition at the lowest concentration) (Fig. 4). Because we did not have access to purified preparations of the *spl*-encoded proteases, we also repeated these experiments using a derivative of LAC capable of producing only the *spl* operon proteases. The presence or absence of spl proteases had no effect on biofilm formation in this assay (Fig. 4). More importantly, these results suggest that the inhibitory effect of each protease is mediated directly by the individual proteases themselves and that aureolysin does in fact have a greater impact on limiting biofilm formation than any of the other extracellular proteases. Additionally, the inhibitory impact of SspA was less apparent in the total protease-deficient derivative of LAC (Fig. 4) than in LAC itself (Fig. 2). Because the wild-type strain produces SspB while the total protease mutant does not, this suggests that the impact of SspA is at least partly due to its impact on activation of SspB.

**Figure 5:**
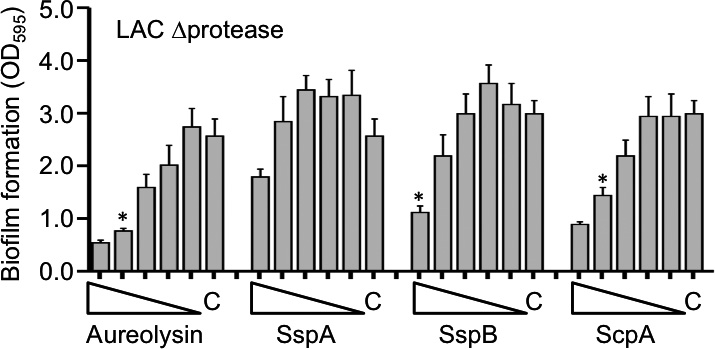
Impact of extracellular purified proteases on dispersal of established biofilms. Biofilms were allowed to form with the LAC protease-deficient mutant for 24 h before adding purified proteases in concentrations ranging from 250 to 16 nmol/L. The impact of each protease on dispersal of the established biofilm was then assessed 24 h later as previously described (Beenken et al. 2010). “C” indicates the control in which no exogenous protease was added. Asterisks indicate the lowest concentration of each protease at which a statistically significant difference was observed relative to this control.

Consistent with these results, we also demonstrated that purified aureolysin had the greatest capacity to promote dispersal of an established biofilm formed by the protease-deficient derivative of LAC (Fig. 5), thus confirming an important role for aureolysin itself rather than its role as a primary mediator of the protease activation cascade. ScpA and SspB were also capable of promoting biofilm dispersal in this strain, while purified SspA had little effect (Fig. 5).

**Figure 6:**
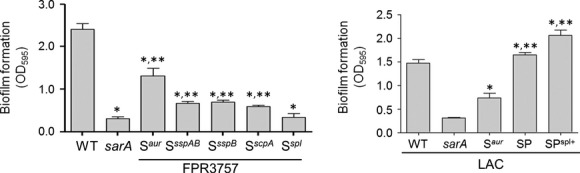
Impact of mutations in individual protease genes/operons on biofilm formation in vitro. The relative capacity to form a biofilm was assessed using a microtiter plate assay as previously described (Beenken et al. 2003) using FPR3757, its *sarA* mutant, and its *sarA* mutant carrying mutations in the indicated protease genes. Single asterisks indicate significance by comparison to the parent strain. Double asterisks indicate significance by comparison to the *sarA* mutant. As a control, biofilm formation was also assessed in LAC, its *sarA* mutant, and derivatives of the *sarA* mutant unable to produce aureolysin (S^*aur*^), unable to produce any extracellular protease (SP), or unable to produce any extracellular protease other than those encoded by the *spl* operon (SP^*spl*+^). Single asterisk indicates statistical significance by comparison to the *sarA* mutant. Double asterisks indicate significance by comparison to the S^*aur*^ mutant.

Taken together, these results suggest that aureolysin, ScpA, and SspB play the most important roles in both biofilm formation and maintenance. To confirm these results, we also took the alternative approach of examining a *S. aureus* strain with mutations inactivating the genes encoding different proteases. Because the mutants were already available and genetically validated, these experiments were done using the USA300 strain FPR3757 and its isogenic *sarA* mutant rather than LAC, but these two USA300 strains have been shown to be essentially identical to each other (Kennedy et al. 2008). Zymogram analysis confirmed the protease phenotypes of these mutants, including the fact that the *sspA* mutant has a polar effect that abolishes the production of both SspA and SspB while the *sspB* mutant abolishes the production of SspB, but has no impact on the production of SspA (Fig. 3). The results also confirmed that mutation of the gene encoding aureolysin (*aur*), the *sspABC* operon, *sspB*, or the *scpAB* operon all enhanced biofilm formation in an FPR3757 *sarA* mutant, with mutation of *aur* having the greatest effect (Fig. 6). In contrast, mutation of the *spl* operon had little impact on biofilm formation in the FPR3757 *sarA* mutant. These results along with those seen in Figure 4 confirmed the same relative effects, suggesting that the *spl*-encoded proteases are unlikely to play a significant role in limiting biofilm formation in *sarA* mutants, at least under in vitro conditions. The observation that mutating the *sspB* gene was not statistically different to that of mutating the *sspABC* operon provides further support for the hypothesis that SspB plays the more important role in limiting biofilm formation by comparison to SspA. However, the impact of mutating each of these genes/operons, while statistically significant, was limited, an observation that provides further support for the hypothesis that multiple extracellular proteases contribute to the biofilm-deficient phenotype of *sarA* mutants (Fig. 6). Further support for this hypothesis comes from the observation that mutation of all of these protease genes was sufficient to fully restore biofilm formation in a LAC *sarA* mutant irrespective of the functional status of the *spl* operon (Fig. 6).

**Figure 7:**
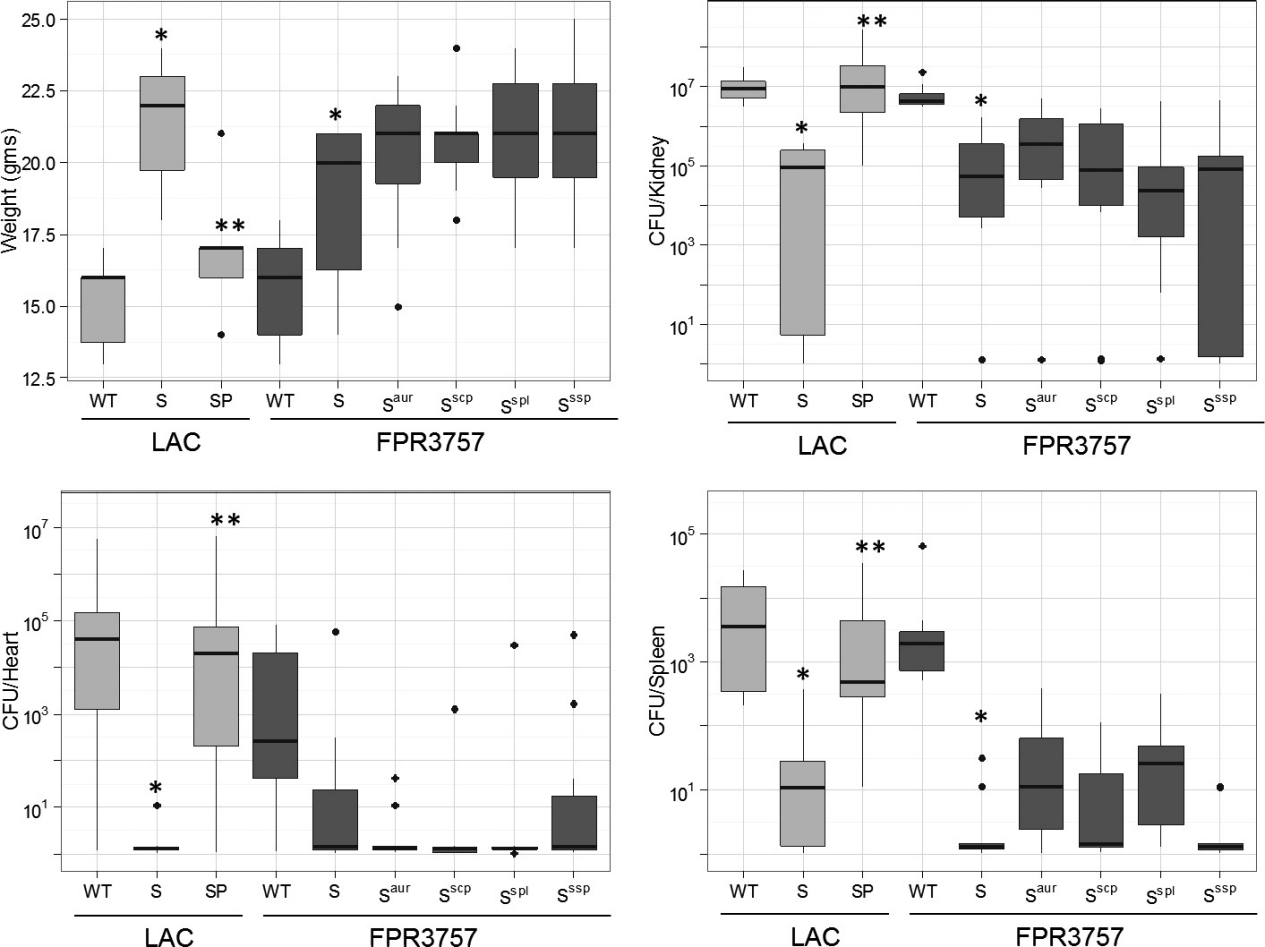
Impact of mutating individual protease genes/operons in vivo. Mice were infected by tail vein injection of LAC, FPR3757, their isogenic *sarA* mutants (S), or *sarA* mutants unable to produce the indicated proteases, with SP indicating a LAC *sarA* mutant unable to produce any extracellular protease. The *ssp* mutant used in these studies is the *sspA* polar mutant unable to produce SspA or SspB. Results shown are weight gain/loss or colony counts in the indicated tissues. Boxes indicate the 25th and 75th percentiles for each group and define the interquartile range (IQR), with the horizontal line indicating the median. Vertical lines define the lowest and highest data points within 1.5 IQR of the lower and higher quartile, respectively, with individual dots representing single data points outside this range. The light gray boxes represent the USA300 strain LAC. The dark gray boxes represent the USA300 strain FPR3757. Single asterisk indicates statistical significance of the *sarA* mutant by comparison to the appropriate parent strain. Double asterisks indicate statistical significance of the sarA mutant by comparison to the isogenic protease-deficient *sarA* mutant.

The hypothesis that multiple proteases are involved is consistent with the observations that individual proteases exhibit differential specificity with respect to *S. aureus* targets previously implicated in biofilm formation (Beenken et al. 2014; Mrak et al.*,* 2012; Zielinska et al. 2011, 2012). However, all of the results discussed above are based on in vitro experiments, leaving open the possibility that they do not accurately reflect in vivo relevance. To address this possibility, we used our murine bacteremia model to examine the impact of eliminating the production of individual proteases in an FPR3757 *sarA* mutant under in vivo conditions, with LAC, its *sarA* mutant, and its *sarA* mutant unable to produce any extracellular protease being included as controls (Zielinska et al. 2012). The results confirmed that mutation of *sarA* attenuates the virulence of both LAC and FPR3757 as assessed by weight loss and bacterial burdens in the kidney, spleen, and heart (Fig. 7). They also confirmed that eliminating the ability of a LAC *sarA* mutant to produce all extracellular proteases reversed this effect. In contrast, eliminating the ability of an FPR3757 *sarA* mutant to produce individual proteases, or in the case of *sspAB* and *splA-F,* multiple proteases encoded within the same operon, had no statistically significant effect. Presumably, this is a reflection of the aforementioned differential specificity of these proteases and its impact on the ability of *S. aureus* to produce multiple virulence factors that contribute to its pathogenesis.

**Figure 8:**
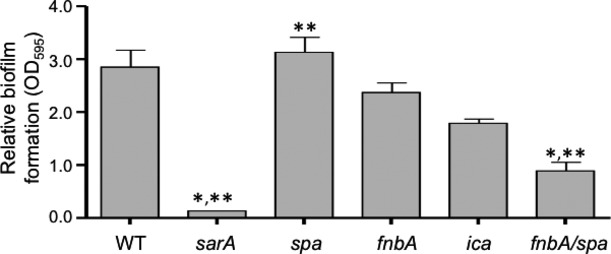
Impact of mutating protease targets genes on biofilm formation. Biofilm formation was assessed in UAMS-1 (WT) and isogenic derivatives with mutations in *sarA*, *spa*, *fnbA*, or *ica*. It should be noted that UAMS-1 does not encode *fnbB* (Cassat et al. 2005). Single asterisk indicates statistical significance by comparison to the parent strain. Double asterisks indicate statistical significance by comparison to the isogenic *ica* mutant.

Finally, to further examine the impact of proteases on *S. aureus* biofilm formation, and specifically address the hypothesis that protein-mediated biofilm formation plays an important role in MRSA, while PIA plays the more important role in MSSA (O'Neill et al. 2008; Houston et al. 2011; Pozzi et al. 2012), we generated mutations in the genes encoding FnbA, Spa, or the enzymes necessary for PIA production (*ica* operon) in the methicillin-susceptible strain UAMS-1 and examined the impact on biofilm formation. The results of these experiments confirmed that protein-mediated biofilm formation plays an important role in this strain and provided a possible explanation for this hypothesis. Specifically, mutation of the *ica* operon had a greater impact in UAMS-1 than mutation of *spa* or *fnbA*. However, mutation of *fnbA* and *spa* limited biofilm formation in UAMS-1 to a significantly greater extent than mutation of *ica* (Fig. 8). These results confirm that protein-mediated biofilm formation is more important than PIA-mediated biofilm formation, even in the methicillin-susceptible strain UAMS-1.

**Figure 9:**
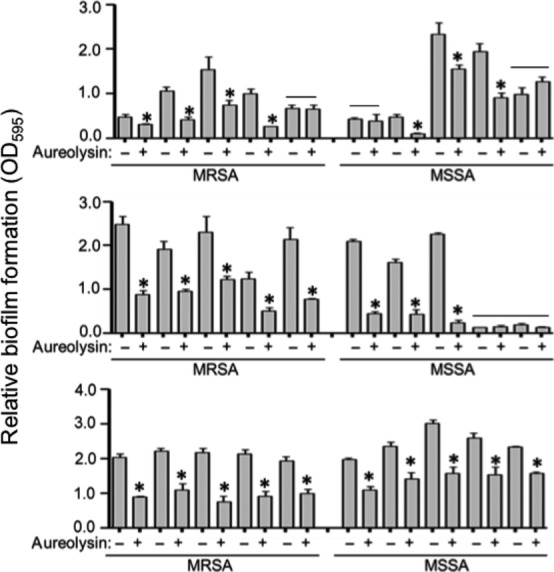
Impact of purified aureolysin in contemporary clinical isolates as a function of methicillin resistance status. A microtiter plate assay was used to assess biofilm formation under standard conditions (−) or after the addition of purified aureolysin at a concentration of 62.5 nM (+). The experiments included 30 primary clinical isolates obtained from the collection at Arkansas Children's Hospital. Asterisks indicate strains in which the addition of aureolysin had a statistically significant impact on biofilm formation. Note that this includes the preponderance of both MRSA and MSSA strains and, conversely, that both groups include a limited number of strains in which biofilm formation was unaffected by the addition of aureolysin (bars). However, these latter strains generally did not form a robust biofilm (OD < 1.0).

**Figure 10:**
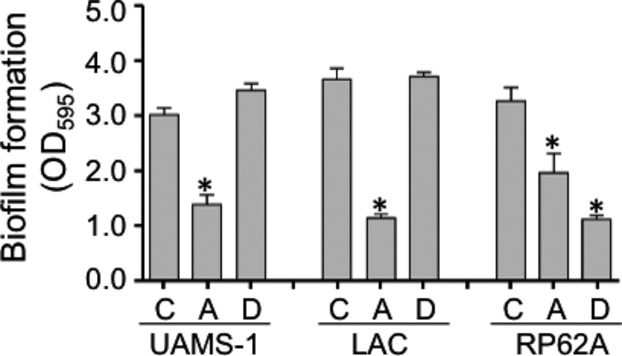
Relative impact of aureolysin and dispersin B on biofilm formation. Biofilm formation was assessed in the *S. aureus* strains UAMS-1 and LAC and the *S. epidermidis* strain RP62A without any additives (C) or after the addition of aureolysin (A) (62.5 nmol/L) or dispersin B (D) (5 *μ*mol/L). Asterisks indicate statistical significance by comparison to the isogenic parent strain.

**Figure 11:**
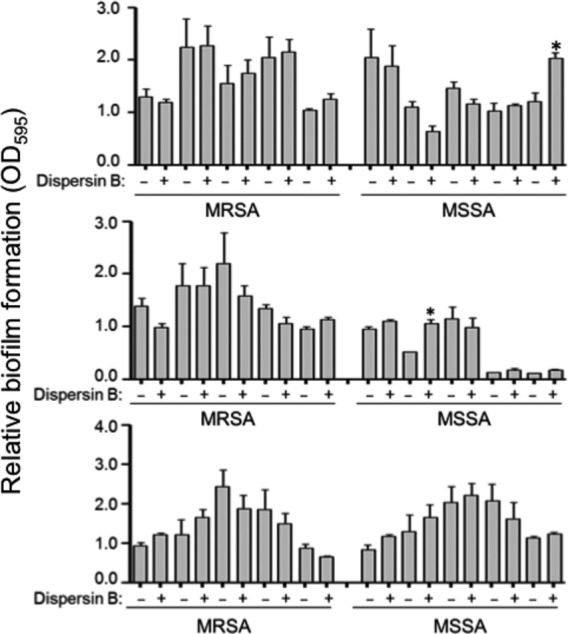
Impact of purified dispersin B in contemporary clinical isolates as a function of methicillin resistance status. A microtiter plate assay was used to assess biofilm formation of the same 30 strains (Fig. 8) under standard conditions (−) or after the addition of purified dispersin B at a concentration of 5 *μ*mol/L (+). Asterisks indicate significant statistical significant by comparison to the isogenic parent strain.

To determine the extent to which this is true in other strains, we evaluated the impact of purified aureolysin on biofilm formation in each of 30 contemporary clinical isolates obtained from different, unrelated patients sourced over a 15-year period at Arkansas Children's Hospital, with these 30 strains being split evenly between MRSA and MSSA. We found that purified aureolysin limited biofilm formation to a statistically significant degree in 14 of 15 MRSA strains and 11 of 15 MSSA strains, with almost all of the exceptions in both cases being strains that did not produce a robust biofilm under the in vitro conditions we employed in our experiments (Fig. 9). In contrast, dispersin B (Kane Biotech), a soluble glycoside hydrolase produced by *Actinobacillus actinomycetemcomitans* that inhibits PIA-mediated biofilm formation (Donelli et al. 2007; Sugimoto et al. 2013), limited biofilm formation in the *S. epidermidis* strain RP62A, but had no impact in UAMS-1, LAC (Fig. 10), or 29 of 30 of these clinical isolates (Fig. 11). Of note is the fact that only one strain was significantly altered by addition of dispersin B but that the change was a significant increase rather than decrease in biofilm formation. Taken together we can show that dispersin B is effective against PIA-mediated biofilm formation, and that methicillin resistance does not play a significant role in determining biofilm structure in clinical isolates.

We conclude from these collective results that extracellular proteases play an important role in limiting biofilm formation in vitro and overall virulence in vivo, but that at least in the context of the attenuation of *sarA* mutants, no single protease can account for these phenotypes. Nevertheless, the results also suggest that aureolysin and the cysteine proteases SspB and ScpA are likely to play the most important roles owing to their differing specificities for specific *S. aureus* proteins that collectively contribute to biofilm formation and virulence. We also conclude that this is likely to be true in diverse contemporary clinical isolates of *S. aureus* irrespective of their methicillin resistance status.
